# Cancer; What Is It? Causes and Treatment

**Published:** 1891-11

**Authors:** C. S. Reeves


For Daniel's Texas Medical Journal

### CANCER; WHAT IS IT? CAUSE AND TREATMENT.

BY C. S. REEVES, M. D.

FORTY years experience in the *art* of medicine (for it is more an art, than a science), has taught me many valuable lessons I could only learn in the school of experience. Theories, opinions and orthodoxy are alike valuable as so many guide posts, in religion, politics and science, but a few *facts* outweigh all of them.

Thirty years ago, or thereabout, the Medical Association of France offered a reward of 30,000 francs for the best written thesis on "The Causes and Treatment of Cancer." A learned trio of the most eminent men in the medical science were selected to act as umpires. During the twelve months preceding their decision, they received a good-sized cart-load of theses; from more than a thousand of these, they selected one, and gave the award to the worthy M. D. who wrote only half a dozen lines, substantially as follows: "The medical world is still at sea on the subject of cancer. There is nothing definite known as to the causes or treatment. We hope that in the not distant future, through the revelations of the microscope, we will know more about it." He received the reward simply, because, like an honest man, he was willing to own or acknowledge the facts in the case. About the same time, the world-renowned surgeon, S. D. Gross, then professor of surgery in the Louisville, Ky., Medical College, stated publicly to his class: "I have operated a great number of times for cancer, because operative surgery is my profession, but, gentlemen, if I had cancer on my little finger I *might*, *perhaps*, allow you to cut it off, but in no case would I allow the use of the knife, were the tumor situated near a vital part, or where it involved any considerable tissue." These are very nearly the exact words of one who stood at the very head of his calling, after growing grayheaded with the surgeon's knife in hand! Can it be surprising that charlatans, mountebanks, quacks and swindlers should take advantage of our want of some system, some correct data, on which to found at least a *theory*?

There are so many sores that simulate cancer, some hereditary and many otherwise, those fellows *call all* of them cancer, and when one is cured, a *certificate* is published to the world, that another cancer has been cured! Again, the doctrine of "heredity" is claimed on the other hand, by all "Regulars" for almost *every chronic* disease they can't cure, and so it comes to pass that "Scylla" is as easily crossed on the one side as "Charibdis" on the other. Apropos, a certain newly-fledged naval surgeon on board of one of the war ships, during our late unpleasantness, having imbibed the doctrine of "heredity" for most of the diseases to which our poor mortality is heir, was suddenly called to attend a sailor, who had a piece of a spar blown through the middle of his body, by the bursting of some part of the engine. Throwing himself on his "shape," and finding the poor man lying on his side in extreme agony, the piece of wood sticking out before and behind, a foot, he said: "You experience great difficulty in attempting to lie on your back, do you?" "Yes, I can't do it at all." "The same trouble in endeavoring to rest on your breast?" "Yes, sir; for God's sake, help me, doctor, if you can!" "Did your father, or mother, or any near relative, ever have a piece of wood stuck through them in this manner?" "No, sir; not that I know of; help!" "Well, it is certain, your disease is not *hereditary*." "Help!" "I could pull out that stick, and you would bleed to death in five minutes; but if I let it remain, you may live an hour." So saying, he walked back to his quarters, and left the poor fellow to his fate.

I had fondly hoped, that through the investigations of Koch and others, something tangible would have developed ere now; and when it was claimed that precisely the same bacteria are found in tuberculosis as in fungous hematodes, I was about to exclaim "Eureka!" But my pen is wandering. "Facts! Facts! Let us have some reliable data;" says the reader. Well, we only have space in this communication to ask a question or two, which we propose to answer at some length in a future letter:

1. Has there *never* been a case of incipient consumption cured?

2. Are there not numerous cases on record, where tubercles of large size have been ejected from the lungs, a cicatrice around the cavity formed, and the patient live in tolerable health for many after years?

3. What about the doctrine of an entire change in the whole animal economy every seven years?

4. Is it not possible, even probable, that (as the lawyers say), to "change the venue" in these cancerous deposits from the blood?

5. Will not the philosophy of *generation* and *regeneration* apply to *infinitesimal animalculæ*, as well as to bipeds of a larger size; and if not, why not?

In conclusion of this article, I respectfully ask my brethren of the ancient order of "powdered wig and gold-headed cane," to withhold their criticism until I am through.

